# Anatomical Resection Improved the Outcome of Intrahepatic Cholangiocarcinoma: A Propensity Score Matching Analysis of a Retrospective Cohort

**DOI:** 10.1155/2022/4446243

**Published:** 2022-10-25

**Authors:** Chao Wang, Pingcuo Ciren, Awang Danzeng, Yong Li, Cheng-Long Zeng, Zhi-Wei Zhang, Zhi-Yong Huang, Yi-Fa Chen, Wan-Guang Zhang, Bi-Xiang Zhang, Bin-Hao Zhang, Xiao-Ping Chen

**Affiliations:** Hepatic Surgery Center, Institute of Hepato-Pancreato-Biliary Surgery, Tongji Hospital, Tongji Medical College, Huazhong University of Science and Technology, Wuhan 430030, China

## Abstract

**Background:**

Intrahepatic cholangiocarcinoma (ICC) is the second most common liver malignancy after hepatocellular carcinoma (HCC), with a dismal prognosis and high heterogeneity. The oncological advantages of anatomical resection (AR) and nonanatomical resection (NAR) in HCC have been studied, but surgical strategies for ICC remain controversial with insufficient investigations.

**Materials and Methods:**

From Jan 2013 to Dec 2016, 3880 consecutive patients were retrospectively reviewed from a single center. Patients with ICC undergoing AR or NAR have been enrolled according to inclusion and exclusion criteria. Propensity score matching (PSM) analysis was performed between two groups with a 1 : 1 ratio. The primary endpoint was overall survival (OS), and the secondary endpoints included disease-free survival (DFS), intraoperative patterns, postoperative morbidity, mortality, complications and recurrence. A prognostic nomogram was developed by a multivariate Cox proportion hazard model.

**Results:**

After PSM, 99 paired cases were selected from 276 patients enrolled in this study. Patients in the AR group achieved better 1-, 3-, and 5-year OS (70%, 46%, and 34%, respectively) and DFS (61%, 21%, and 10%, respectively) than patients in the NAR group with statistical significance after PSM analysis. The postoperative complications and recurrence patterns were comparable between the two groups. Multivariate analysis identified NAR, tumor size >5 cm, multiple tumors, and poor differentiation as independent risk factors for OS (*p* < 0.05). Selected patients can benefit most from AR, according to subgroup analysis. A prognostic nomogram based on six independent risk factors for OS and factors with clinical significance was constructed to predict OS in ICC patients.

**Conclusion:**

AR improved the long-term survival of ICC with comparable postoperative complications and similar recurrence patterns. AR is suggested in ICC patients with sufficient remnant liver volume. In addition to surgery strategy, malignant characteristics of tumors are risk factors for ICC prognosis.

## 1. Introduction

Cholangiocarcinoma (CCA) is a highly lethal hepatic malignancy with increasing incidence and mortality worldwide, with 0.3–6 cases per 100,000 inhabitants per year in Western and over 6 cases in some East Asian regions [1–3]. This heterogeneous cancer with aggressive invasiveness can be classified as intrahepatic CCA (iCCA or ICC), perihilar CCA (pCCA), or distal CCA (dCCA), according to the anatomic structure [4–6]. Though advances in neoadjuvant chemotherapy and targeting immunotherapy have brought scientific and clinical insights into treatments for ICC, there is still a lack of decisive evidence for their clinical application [7, 8].

Surgical resection still remains the only potentially curative treatment, with low resectability rates varying from 19% to 74% [9]. The surgery strategies and resection range are controversial for ICC in speaking of prognosis improvement. Resection margin and lymph node metastasis are significant prognostic factors, but it is debated whether wide resection margins or lymphadenectomy improved postoperative survival [10, 11]. Aggressive invasiveness characteristics of ICC present multifocality, lymph node metastasis, and vascular invasion with dismal outcomes [12]. The optimal therapeutic strategy for ICC has not been determined yet [13]. Appropriate preoperative surgical planning and subsequent treatments decided by multidisciplinary team (MDT) discussion are indispensable in achieving optimal outcomes for ICC patients [13].

Anatomical resection (AR) was firstly proposed by Japanese surgeon Makuuchi in the 1980s to eradicate potential micrometastasis [14]. Studies for oncological advantages of AR in primary liver cancer mainly focus on hepatocellular carcinoma (HCC), though the results have been long controversial [15, 16]. Compared with intrahepatic metastasis of HCC via vascular structures, tumor cells of ICC spread along with the biliary tree and lymph system, which supports the oncological and prognostic superiority of AR over nonanatomical resection (NAR) in theory [17]. The complete excision of tumor-bearing portal tributaries of AR in ICC may prevent microscopic intrahepatic metastasis by preventing tumor cells from spreading along the biliary tree or lymph system and reducing the rates of local recurrence and prolonging survival. The efficacy of AR for ICC may also vary according to clinicopathological factors, including tumor size, number, location, differentiation, preoperative liver function, and remnant liver parenchyma.

However, the impact of AR and NAR on short-term and long-term outcomes in ICC still lacks investigation [18–20]. We conducted this retrospective study to evaluate the operative and postoperative characteristics of AR and NAR groups. Propensity score matching (PSM) analysis was applied to minimize the selection bias of the surgery strategy.

## 2. Materials and Methods

### 2.1. Patient Enrollment

From Jan 2013 to Dec 2016, 3880 consecutive patients were retrospectively reviewed in the Institute of Hepato-Pancreato-Biliary Surgery, Tongji Hospital, Tongji Medical College, Huazhong University of Science and Technology. The inclusion criteria were as follows: (1) patients with ICC confirmed pathologically; (2) patients with age ≥18 years; (3) patients initially treated with AR or NAR; (4) patients with R0 resection margin. The exclusion criteria were as follows: (1) patients with severe underlying diseases; (2) patients with other malignancies; (3) patients with metastasis prior to the resection; (4) patients who received previous antitumor treatments; (5) patients undergoing hepatectomy combined with transarterial chemoembolization (TACE) or ablation; (6) patients with microscopically positive (R1) resection or macroscopically positive (R2) resection margin; (7) patients who died within 30 days after surgery or due to other nontumor causes; (8) patients with insufficient clinical data.

This study was reviewed and approved by the Medical Ethics Committee of Tongji Hospital, Tongji Medical College, Huazhong University of Science and Technology. Written informed consent was obtained from the patients.

### 2.2. Liver Resection and Follow-Up

Surgery strategy of AR and NAR was determined based on a general evaluation of jaundice, serum level of total bilirubin, and remnant liver volume assessed by CT scanning and three-dimensional reconstruction techniques as well as indocyanine green retention at 15 min (ICGR15). AR was defined as the complete resection of Couinaud's segments by prior ischemia or dye staining of indocyanine green (ICG) fluorescence, including segmentectomy, lobectomy, or hemihepatectomy. NAR was defined as incomplete resection of the portal tributaries of the lesion segment with a surgical margin of at least 1 cm or without exposing the tumor surface on the parenchymal transection, including partial resection or enucleation of the liver tumor [15, 17, 21]. Intraoperative ultrasound was routinely performed to evaluate tumor location, number, size and adjacent parenchyma, in addition to preoperative imaging. Pringle's maneuver was routinely performed with interval clamping/unclamping of 10 min/5 min. Portal occlusion and/or inferior vena cava (IVC) occlusion were applied when necessary. Routine lymphadenectomy at the level of hepato-duodenal ligament has been performed according to guidelines [22]. All patients in this study have achieved R0 resection.

The first follow-up was carried out 1 month after the operation, and every 2-3 months within the first year, then every 6–12 months afterwards. Physical examination and measurements of liver and kidney function, serum level of tumor markers (carbohydrate antigen 19–9 (CA19-9), carcinoembryonic antigen (CEA), and alpha-fetoprotein (AFP)), and imaging examination (abdominal ultrasound, contrast-enhanced computed tomography (CT), magnetic resonance imaging (MRI), and chest X-ray) were performed during the follow-up. When recurrence occurred during the follow-up, reoperation, microwave ablation, transarterial chemoembolization (TACE), chemotherapy, radiotherapy, targeted therapy, immunotherapy or palliative care were given according to clinical guidelines, MDT discussion, and the patients' wishes [6, 22–25]. The last follow-up date was Jan 2022.

### 2.3. Study Endpoints

The primary endpoint was overall survival (OS). The secondary endpoints included disease-free survival (DFS), intraoperative parameters (operation time, blood loss, blood transfusion, portal vein/IVC occlusion and laparoscopic/open surgery), postoperative morbidity, mortality, complications, and recurrence (intrahepatic, extrahepatic, or both). OS was defined as the time from the date of surgery to either the date of death or the last date of follow-up, while DFS was defined as the time from the date of surgery to either the date of disease recurrence or the last date of follow-up. Postoperative morbidity and mortality were defined as events that happened within the first 60 days after surgery. Complications were graded according to the Clavien–Dindo classification [26].

### 2.4. Propensity Score Matching Analysis and Nomogram Prediction

PSM analysis was introduced to reduce the bias of treatment selection. As previously described, the ICC patients in AR and NAR groups were matched by propensity score [27]. The propensity score for an individual was calculated given the covariates of tumor number, Child–Pugh classification, lymph node metastasis, and portal vein tumor thrombosis (PVTT) in pathology using a logistic regression model. Thereafter, 1 : 1 nearest neighbor matching with a calliper of 0.01 and without replacement was applied to ensure minimized conditional bias [28].

Independent risk factors selected by multivariate analysis for OS and the demographic characteristics with clinical significance were incorporated into the nomogram to predict 1-, 3-, and 5-year survival.

### 2.5. Statistical Analysis

Categorical variables are presented as numbers (%) and were compared by the Chi-squared test. Continuous variables are presented as mean ± standard deviation (SD). Normally distributed data were compared with Student's *t*-test, and nonnormally distribution was analyzed by the Mann–Whitney *U* test. The Kaplan–Meier method was applied to depict the survival curves before and after PSM, compared using the Log-rank test. Candidate variables with statistical significance in univariate analysis were introduced into multivariable Cox proportional hazards regression analyses to determine the independent risk factors associated with OS and DFS in the PSM cohort. In order to evaluate the impact of surgical strategy (AR or NAR) on OS, the groups were included in the multivariable Cox regression, regardless of whether the *p* value was statistically significant in univariate analysis. Statistical analysis was performed by IBM SPSS version 22.0 (SPSS Inc., Chicago, IL, USA) and R software version 4.1.2 with the “rms,” “survival,” “survminer,” “ggsci,” and “forestplot” packages. *p* < 0.05 was considered to be statistically significant.

## 3. Results

### 3.1. Perioperative Characteristics

**Figure 1:**
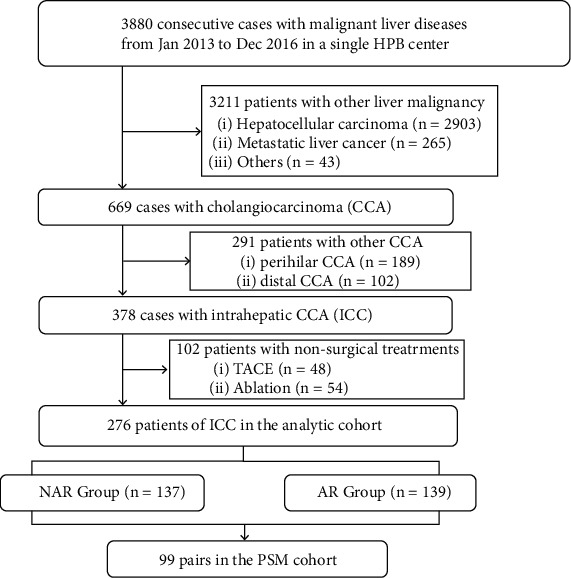
The flowchart of patient enrollment in this study.

From Jan 2013 to Dec 2016, a total of 3880 consecutive patients with malignant liver diseases from our single HPB center were retrospectively reviewed and validated. In these cases, patients with hepatocellular carcinoma (*n* = 2903), metastatic liver cancer (*n* = 265) and other malignancies (*n* = 43) were excluded. Among the patients with cholangiocarcinoma (*n* = 669), patients with perihilar CCA (*n* = 189) and distal CCA (*n* = 102) and patients undergoing TACE (*n* = 48) and microwave ablation (*n* = 54) were excluded. 276 ICC patients with surgical treatment were initially included in the analytic cohort, with 137 patients in the NAR group and 139 patients in the AR group. PSM analysis determined 99 pairs of patients for further survival and risk factor analysis (Figure 1).

Perioperative characteristics are summarized in Table 1. The preoperative RBC count was lower in the AR group (*p*=0.038). There were more patients in the NAR group with Child–Pugh class A (91.97% vs. 84.17%), compared with those in the AR group (*p*  = 0.046). There is no significant difference among the other baseline characteristics in two groups. The operation time in the AR group was longer (*p*=0.028). Patients in the AR group had a larger amount of intraoperative hemorrhage (*p*=0.045) and the percentage of transfusion was higher (*p*=0.023). More patients in AR group had singular lesion (*p*=0.001), lymph node metastasis (*p*=0.006) and PVTT (*p*=0.033). The hospital stay was shorter in NAR group (*p* < 0.001). There was no statistical significance in the other operative and postoperative parameters between the two groups.

PSM analysis selected 99 matched pairs from each group. There were no significant differences in baseline characteristics in AR and NAR groups after PSM. However, more patients in the AR group had transfusions (*p*=0.009), and the hospital was longer in the AR group (*p*=0.008) as well. There were no significant differences in the incidence of complication (*p*=0.534), types (*p*=0.882), or Clavien–Dindo grade (*p*=0.825) between the two groups in the PSM cohort (Table 2).

### 3.2. Survival Analysis

**Figure 2:**
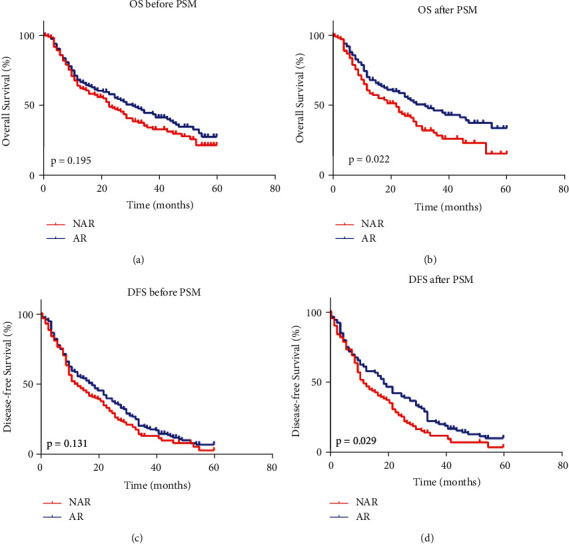
Kaplan–Meier curves of overall survival and disease-free survival in ICC patients before and after PSM. (a) overall survival of ICC patients before PSM; (b) overall survival of ICC patients after PSM; (c) disease-free survival of ICC patients before PSM; (d) disease-free survival of ICC patients after PSM.

Before PSM analysis, the 1-, 3-, and 5-year OS in the AR group (68%, 44%, and 28%, respectively) were comparable to those in the NAR group (64%, 34%, and 22%, respectively), though the Kaplan–Meier curves were suggestive but not significant (Figure 2(a)). There was no significant difference of 1-, 3-, and 5-year DFS between AR group (58%, 19%, and 7%, respectively) and NAR group (50%, 13%, and 3%, respectively) (Figure 2(c)). Patients showed better 1-, 3-, and 5-year OS in AR group (70%, 46%, and 34%, respectively) than NAR group (60%, 28%, and 16%, respectively) (*p*=0.022) (Figure 2(b)) after PSM analysis. In the PSM cohort, the 1-, 3-, and 5-year DFS in the AR group (61%, 21%, and 10%, respectively) were significantly better than those in the NAR group (49%, 12%, and 4%, respectively) (*p*=0.029) (Figure 2(d)).

During the follow-up, 6 (6.06%) patients in the AR group and 1 (1.01%) patient in the NAR group did not report disease recurrence (Table 3). Recurrence pattern analysis showed there were no statistical differences in intrahepatic recurrence, extrahepatic recurrence, and both intra-/extrahepatic recurrence. Patients in the AR group tended to have an intrahepatic recurrence in distant segments (39.39% vs. 31.31%) and less possibility of recurrence in resection margin (2.02% vs. 5.05%) or adjacent segments (26.26% vs. 32.32%), though the difference was not statistically significant (*p*=0.245).

**Figure 3:**
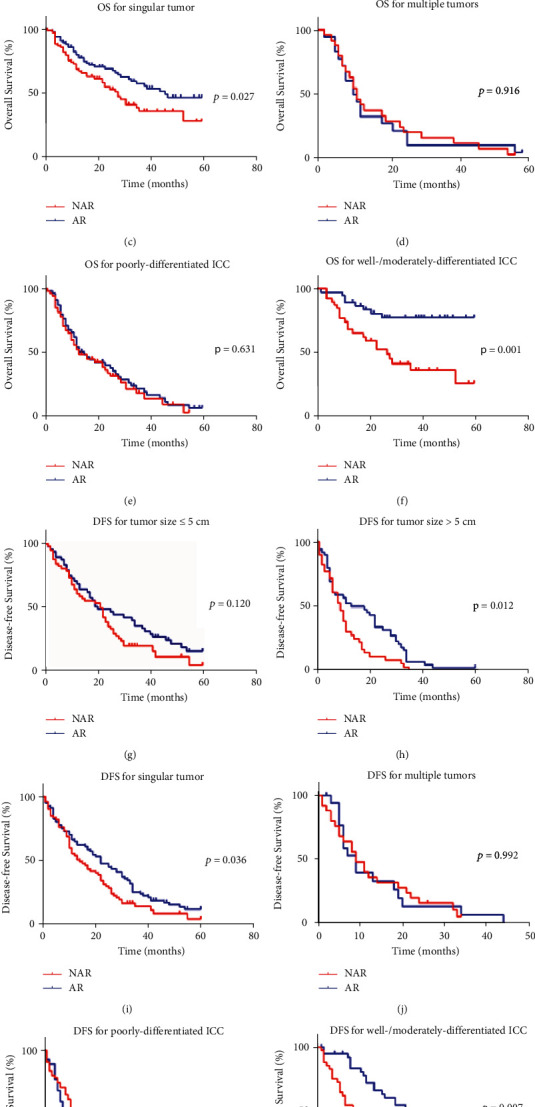
Kaplan–Meier curves of subgroup analysis of overall survival and disease-free survival in ICC patients who underwent AR or NAR. (a) overall survival of patients with tumor ≤5 cm; (b) overall survival of patients with tumor >5 cm; (c) overall survival of patients with singular tumor; (d) overall survival of patients with multiple tumors; (e) overall survival of patients with poor differentiation; (f) overall survival of patients with well/moderate differentiation; (g) disease-free survival of patients with tumor ≤5 cm; (h) disease-free survival of patients with tumor >5 cm; (i) disease-free survival of patients with singular tumor; (j) disease-free survival of patients with multiple tumors; (k) disease-free survival of patients with poor differentiation; (l) disease-free survival of patients with well/moderate differentiation.

**Figure 4:**
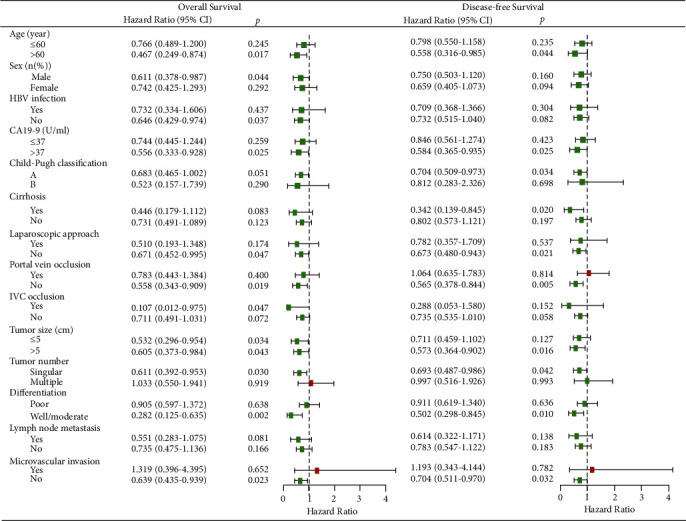
Forest plot for subgroup analysis of overall survival and disease-free survival in two groups after PSM.

Subgroup analysis of OS and DFS after PSM analysis showed that the surgical strategy of AR in selected patients had an associated improved prognosis (Figure 3). Specific subgroups of patients, age >60 years, male, without HBV infection, CA19-9 > 37 U/mL, without laparoscopic approach, without portal vein occlusion, with IVC occlusion, singular tumor, well/moderate differentiation, without microvascular invasion, regardless of the tumor size, may benefit more from AR in OS. Other subgroups of patients, age >60 years, CA19-9 > 37 U/mL, Child–Pugh class A, with cirrhosis, without laparoscopic approach, without portal occlusion, tumor size >5 cm, singular tumor, well/moderate differentiation, and without microvascular invasion, may benefit more from AR in DFS (Figure 4).

### 3.3. Risk Factor Analysis

In the PSM cohort, the univariate analysis identified NAR, tumor size >5 cm, multiple tumors, poor differentiation and lymph node metastasis as significant risk factors for OS (*p* < 0.05) (Table 4). NAR, without laparoscopic approach, tumor size >5 cm, multiple tumors, poor differentiation and lymph node metastasis were considered significant risk factors for DFS (*p* < 0.05) (Table 5). Multivariate analysis showed NAR, tumor size >5 cm, multiple tumors and poor differentiation were independent risk factors for OS (*p* < 0.05), NAR, tumor size >5 cm, and poor differentiation were independent risk factors for DFS (*p* < 0.05) after PSM analysis.

### 3.4. Nomogram Prediction

**Figure 5:**
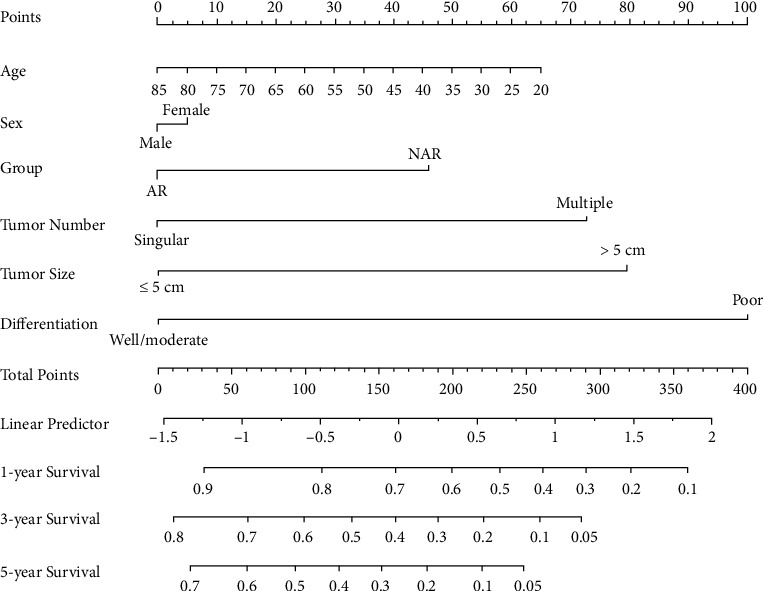
Nomogram for postoperative survival prediction based on six prognostic factors.

A nomogram model predicting OS of patients with ICC undergoing hepatectomy is shown in Figure 5. The prognostic nomogram was developed based on the following six prognostic factors: age, sex (male or female), group (AR or NAR), tumor number (singular or multiple), tumor size (≤5 cm or >5 cm), and differentiation (well/moderate or poor). Each factor was ascribed a weighted point total that implied a survival prognosis.

## 4. Discussion

ICC is the second most common primary hepatic malignant tumor, with radical liver resection as the only curative option [29]. Extended liver resection and vascular reconstruction, together with systemic therapy and locoregional treatments, enabled increasing rates of resection and improved OS in selected ICC patients [30]. AR and NAR have been debated in HCC for decades with still controversial results, while clinical studies focusing on surgical strategy of AR or NAR for ICC are rare. In this study, we found that AR improved the 1-, 3-, and 5-year OS (70%, 46%, and 34%, respectively) and DFS (61%, 21%, and 10%, respectively) for ICC patients with statistical significance after PSM analysis. AR presented comparable complications and recurrence when compared with NAR. Multivariate analysis identified NAR, tumor size >5 cm, multiple tumors, and poor differentiation as independent risk factors for OS (*p* < 0.05). Selected patients can benefit most from AR, according to subgroup analysis. A nomogram based on independent risk factors for OS and factors with clinical significance was constructed to predict OS in ICC patients.

Primary liver cancer is the fifth most commonly diagnosed malignancy worldwide, with a high prevalence in Asia and Africa. HCC arising from hepatocytes and ICC from bile duct epithelium are major types of primary liver cancer [31, 32]. ICC is located in the second-order bile ducts in the hepatic parenchyma, extinguished with pCCA in left and right common hepatic ducts and dCCA in common bile duct [33]. ICC can be classified as mass-forming, periductal infiltrating, and intraductal growth types by morphology [34, 35]. Chronic biliary tract inflammation owing to choledocholithiasis, cholelithiasis, primary sclerosing cholangitis, or liver fluke infection is associated with CCA, while patients are usually asymptomatic and have no underlying liver diseases [36]. Patients with HCC usually have underlying diseases like HBV/HCV infection, steatohepatitis or cirrhosis, and inclined to metastasis with blood flow, while ICC characterized itself with jaundice caused by biliary obstruction and lymph node metastasis. HBV/HCV infection may also be involved in the carcinogenesis of ICC [37, 38]. Interestingly, HBV-associated ICC has been reported to have a favorable prognosis, probably due to early diagnosis [39, 40]. Capecitabine is now first-line adjuvant therapy after curative intent resection [29]. Consecutive therapy plans based on MDT discussion are needed for this aggressive cancer with distinct anatomic, molecular, and clinical characteristics [41, 42].

Radical surgical treatment is still the only therapy with curative potential for ICC. An aggressive surgical approach, including major liver resection, has been recommended in many centers to improve outcomes. Our study found that patients with ICC benefited from AR in OS and DFS after PSM, indicating complete removal of tumor-bearing segments plays a significant role in improving the survival outcomes. Shen reported better survival outcomes were associated with AR in ICC patients with stage IB or II tumors without vascular invasion [18]. The 1-, 3-, and 5-year OS were 72.9%, 45.7%, and 36.0% in the AR group and 62.0%, 30.8%, and 25.3% in the NAR group after PSM. However, Yang concluded that NAR was not inferior to AR in survival outcomes for primary solitary ICC without direct invasion of contiguous organs or extrahepatic metastasis and potential benefits exist in NAR [19]. In their study, the NAR group had a more positive surgical margin, but the surgical margin had no significant impact on OS or DFS before and after PSM analysis.

Resection margin status and length are supposed to be associated with the incidence of local recurrence in theory, which is a technical concern during ICC surgery [11]. Technically, AR obtains a larger distance between surgical margin and tumor lesion, which had a higher potential for negative resection margin than NAR. Previous studies found a residual tumor on the surgical margin is likely to grow and spread much more aggressively, leading to early recurrence and dismal survival [43, 44]. However, the prognostic value of a wide margin remains controversial [10]. Resection margin ≥1 cm was associated with improved survival. Intrahepatic recurrence is inclined to happen in resection margin or adjacent segments in the NAR group but distant segments in the AR group. The result was suggestive based on proportion, although there was no significant difference. Postoperative complications were comparable (Clavien–Dindo grade I/II) in two groups, indicating both AR and NAR are technically safe in hepatectomy for ICC. Though surgical treatments improve the survival of ICC patients, systematic surveillance among patients with high-risk factors is necessary to avoid a late diagnosis of ICC in intermediate/advanced stages [33].

Lymph node (LN) metastases are recognized as an extremely poor prognostic risk factor no matter whether curative resection is applied [34]. The essential of surgery in patients with LN metastases detected preoperatively and routine lymphadenectomy in ICC surgery remain controversial [45]. The 8^th^American Joint Committee on Cancer (AJCC) staging system recommends six nodes need to be analyzed. Combined with resection margin and perineural invasion, lymph node ratio (LNR) 15 was reported to be an independent predictor of DFS, OS, early, local, and distal recurrence [46]. In our research, LN metastases were a relative risk factor for OS in univariate analysis, but it was not an independent risk factor in multivariate analysis. However, lymphadenectomy is still recommended in ICC patients undergoing hepatectomy by experienced surgeons with/without preoperation evidence of LN metastasis [22, 47]. Accurate staging determined by routine lymphadenectomy is crucial for predicting prognosis and providing options for following treatments [48]. In addition, complete removal of lymph nodes with metastasis potential helps to reduce rates of regional recurrence, as well as jaundice or pyloric obstruction induced by recurrence.

The diagnosis of cholangiocarcinoma is accomplished by the combination of clinical/biochemical features and imaging findings with nonspecific tumor markers for suggestive complementary [49]. There is a lack of prognostic and predictive tumor markers for ICC, particularly compared with the clinical significance indicated by elevated AFP level and prognostic efficiency of PIVKA-II in HCC [50, 51]. The combination of CEA, CA125, and CA19-9 had been reported to have diagnostic effects [52, 53]. In our research, about half of the patients presented elevated CA19-9 before or after PSM analysis, while only a very small proportion of patients presented abnormal serum CEA and AFP. In subgroup analysis, AR was superior to NAR in patients with elevated CA19-9, which shows low sensitivity in early stages but increased sensitivity in advanced diseases [2, 54]. Besides traditional serum biomarkers, biomarkers from extracellular vesicles, metabolites, and nucleic acids, as well as next-generation biomarkers detected by high-throughput omics-based approaches, have the clinical application potential [49, 55].

Although surgical resection is the only curative treatment for patients with ICC, most patients are ineligible for surgery treatment at the time of the first diagnosis due to metastasis or local advancement [36]. Early detection of ICC by screening is of vital importance in potential population with risk factors. Tumor size, R0 resection, lymph node metastasis, differentiation, adjuvant chemotherapy, CA19-9, T stage, PVTT, HBV infection/vaccination, and Eastern Cooperative Oncology Group performance status (ECOG-PS) have been reported as prognostic factors influencing survival outcomes of ICC in recent researches [2, 31, 37, 40, 56–58]. We identified NAR, tumor size >5 cm, multiple tumors, and poor differentiation as independent risk factors for OS in ICC patients undergoing hepatectomy. The nomogram in our study was developed from independent risk factors and factors with clinical significance. The AJCC TNM system is the most commonly used staging system for ICC, while other systems have been proposed, including the staging system for mass-forming type by Okabayashi and the LCSGJ system by Wang. [59]. Many researchers have attempted to establish nomogram models to be used as alternative standards in staging ICC subgroups [59–61].

The minimally invasive approach for curative surgery of ICC has not been well established [62]. Laparoscopy is associated with less intraoperative blood loss, faster recovery, and fewer complications for most surgeries in general, but the benefits of laparoscopic resection for ICC are controversial due to the challenges in major hepatectomy, vascular and biliary reconstruction, and extended lymphadenectomy. Complete resection (R0) with adequate remnant liver parenchyma is the aim of resection regardless of surgery types, which should be technically feasible for both open and minimally invasive approaches. We enrolled 19 patients and 17 patients in the PSM cohort undergoing NAR or AR with a laparoscopic approach, respectively. Without laparoscopic approach was considered a risk factor for DFS in univariate analysis, though multivariate analysis showed it cannot predict the prognosis independently. Subgroup analysis showed AR and NAR were comparable in the laparoscopic group, while AR achieved better OS in patients with open surgery, which might be the result of patient selection. Surgeons preferred to choose laparoscopy on patients with singular, relatively smaller tumors, and better general situation to ensure the operation safety, the outcomes of whom tended to be impacted more by tumor characteristics rather than surgical intervention. However, the advantages of AR can be observed in patients with open surgery. Robotic resection has not been further analyzed due to the limited cases in the entire cohort (2.92% and 2.28%) and in the PSM cohort (3.03% and 3.04%) for NAR and AR groups.

We acknowledge the potential limitations in this study of selection bias and sample size. Although PSM analysis was applied to reduce the selection bias in a new cohort with comparable baseline characteristics, the possibility of other unconsidered biases remains in a retrospective study. The entire cohort was selected from 3880 consecutive patients from a single center in the past 4 years according to the inclusion and exclusion criteria; however, further randomized clinical trials (RCTs) among multiple centers with large surgery volumes are still demanded.

In conclusion, AR improved the long-term survival of ICC with comparable postoperative complications and similar recurrence patterns. Multivariate analysis showed NAR, tumor size >5 cm, multiple tumors, and poor differentiation were independent risk factors for OS. AR is suggested in ICC patients with sufficient remnant liver volume.

## Figures and Tables

**Table 1:** Characteristics of the patients in the two groups before and after PSM.

	Before propensity matching			After propensity matching		
	NAR (*n* = 137)	AR (*n* = 139)	*p*	NAR (*n* = 99)	AR (*n* = 99)	*p*
Age (year) (*n* (%))			0.496			0.289
≤60	94 (68.61%)	90 (64.75%)		70 (70.71%)	63 (63.64%)	
>60	43 (31.39%)	49 (35.25%)		29 (29.29%)	36 (36.36%)	

Sex (*n* (%))			0.826			0.885
Male	82 (59.85%)	85 (61.15%)		58 (58.59%)	59 (59.60%)	
Female	55 (40.15%)	54 (38.85%)		41 (41.41%)	40 (40.40%)	

HBV infection (*n* (%))			0.842			0.605
Yes	36 (26.28%)	38 (27.34%)		20 (20.20%)	23 (23.23%)	
No	101 (73.72%)	101 (72.66%)		79 (79.80%)	76 (76.77%)	

Smoke history (*n* (%))			0.731			0.874
Yes	39 (28.47%)	37 (26.62%)		28 (28.28%)	27 (27.27%)	
No	98 (71.53%)	102 (73.38%)		71 (71.72%)	72 (72.73%)	

Alcohol history (*n* (%))			0.831			0.849
Yes	25 (18.25%)	24 (17.27%)		17 (17.17%)	16 (16.16%)	
No	112 (81.75%)	115 (82.73%)		82 (82.83%)	83 (83.84%)	

RBC count (10^12^/L)	4.37 ± 0.58	4.24 ± 0.52	0.038	4.35 ± 0.60	4.25 ± 0.51	0.209
HB (g/L)	129.52 ± 19.68	128.11 ± 18.10	0.535	128.59 ± 19.73	127.92 ± 17.94	0.803

ALT (U/L) (*n* (%))			0.189			0.865
≤40	107 (78.10%)	99 (71.22%)		77 (77.78%)	76 (76.77%)	
>40	30 (21.90%)	40 (28.78%)		22 (22.22%)	23 (23.23%)	

AST (U/L) (*n* (%))			0.194			0.736
≤40	106 (77.37%)	98 (70.50%)		77 (77.78%)	75 (75.76%)	
>40	31 (22.63%)	41 (29.50%)		22 (22.22%)	24 (24.24%)	

ALB (g/L) (*n* (%))			0.207			0.340
≤35	18 (13.14%)	26 (18.71%)		14 (14.14%)	19 (19.19%)	
>35	119 (86.86%)	113 (81.29%)		85 (85.86%)	80 (80.81%)	
TB (*μ*mol/L) (*n* (%))			0.341			0.728
≤20.5	110 (80.29%)	105 (75.54%)		77 (77.78%)	79 (79.80%)	
>20.5	27 (19.71%)	34 (24.46%)		22 (22.22%)	20 (20.20%)	
BUN (mmol/L)	4.84 ± 1.41	4.85 ± 1.62	0.961	4.82 ± 1.42	4.84 ± 1.54	0.917
CR (*μ*mol/L)	68.30 ± 16.72	68.23 ± 15.83	0.972	68.08 ± 15.16	69.16 ± 16.09	0.627
PT (s)	13.85 ± 1.02	13.75 ± 1.10	0.424	13.90 ± 1.05	13.74 ± 1.08	0.291
PTA (%)	90.37 ± 13.36	92.39 ± 14.57	0.232	89.71 ± 13.64	92.62 ± 14.05	0.141
APTT (s)	37.27 ± 3.57	37.80 ± 4.81	0.302	37.33 ± 3.63	37.67 ± 4.41	0.556

Child–Pugh classification (*n* (%))			0.046			0.817
A	126 (91.97%)	117 (84.17%)		88 (88.89%)	89 (89.90%)	
B	11 (8.03%)	22 (15.83%)		11 (11.11%)	10 (10.10%)	

AFP (ng/mL) (*n* (%))			0.838			0.470
≤40	128 (93.43%)	129 (92.81%)		94 (94.95%)	96 (96.97%)	
>40	9 (6.57%)	10 (7.19%)		5 (5.05%)	3 (3.03%)	

CA19-9 (U/mL) (*n* (%))			0.396			0.668
≤37	75 (54.74%)	69 (49.64%)		56 (56.57%)	53 (53.54%)	
>37	62 (45.26%)	70 (50.36%)		43 (43.43%)	46 (46.46%)	

CEA (ng/mL) (*n* (%))			0.682			0.579
≤20	127 (92.70%)	127 (91.37%)		91 (91.92%)	93 (93.94%)	
>20	10 (7.30%)	12 (8.63%)		8 (8.08%)	6 (6.06%)	

Cirrhosis (*n* (%))			0.508			0.576
Yes	28 (20.44%)	33 (23.74%)		19 (19.19%)	16 (16.16%)	
No	109 (79.56%)	106 (76.26%)		80 (80.81%)	83 (83.84%)	

Laparoscopic approach (*n* (%))			0.313			0.712
Yes	27 (19.71%)	21 (15.11%)		19 (19.19%)	17 (17.17%)	
No	110 (80.29%)	118 (84.89%)		80 (80.81%)	82 (82.83%)	

Robotic approach (*n* (%))			0.983			0.700
Yes	4 (2.92%)	4 (2.88%)		3 (3.03%)	4 (4.04%)	
No	133 (97.08%)	135 (97.12%)		96 (96.97%)	95 (95.96%)	

Portal vein occlusion (*n* (%))			0.054			0.054
Yes	41 (29.93%)	57 (41.01%)		29 (29.29%)	42 (42.42%)	
No	96 (70.07%)	82 (58.99%)		70 (70.71%)	57 (57.58%)	

IVC occlusion (*n* (%))			0.813			0.516
Yes	7 (5.11%)	8 (5.76%)		4 (4.04%)	6 (6.06%)	
No	130 (94.89%)	131 (94.24%)		95 (95.96%)	93 (93.94%)	

Operation time (min)	134.080 ± 112.790	167.990 ± 141.030	0.028	143.420 ± 122.812	165.400 ± 135.210	0.233

Intraoperative hemorrhage (mL)	272.800 ± 257.337	372.900 ± 473.712	0.045	266.230 ± 266.020	375.220 ± 532.383	0.094

Intraoperative transfusion (*n* (%))			0.023			0.009
Yes	7 (5.11%)	18 (12.95%)		3 (3.03%)	13 (13.13%)	
No	130 (94.89%)	121 (87.05%)		96 (96.97%)	86 (86.87%)	

Differentiation (*n* (%))			0.822			0.885
Poor	78 (56.93%)	81 (58.27%)		57 (57.58%)	58 (58.59%)	
Well/moderate	59 (43.07%)	58 (41.73%)		42 (42.42%)	41 (41.41%)	

Tumor size (cm)			0.225			0.153
≤5	78 (56.93%)	69 (49.64%)		60 (60.61%)	50 (50.51%)	
>5	59 (43.07%)	70 (50.36%)		39 (39.39%)	49 (49.49%)	

Tumor number			0.001			0.228
Singular	85 (62.04%)	112 (80.58%)		74 (74.75%)	80 (80.81%)	
Multiple	52 (37.96%)	27 (19.42%)		25 (25.25%)	19 (19.19%)	

Lymph node metastasis			0.006			0.127
Yes	28 (20.44%)	49 (35.25%)		27 (27.27%)	18 (18.18%)	
No	109 (79.56%)	90 (64.75%)		72 (72.73%)	81 (81.82%)	

Neuron invasion			0.130			0.700
Yes	3 (2.19%)	8 (5.76%)		3 (3.03%)	4 (4.04%)	
No	134 (97.81%)	131 (94.24%)		96 (96.97%)	95 (95.96%)	
Necrosis			0.419			0.312
Yes	2 (1.46%)	4 (2.88%)		1 (1.01%)	3 (3.03%)	
No	135 (98.54%)	135 (97.12%)		98 (98.99%)	96 (96.97%)	

PVTT			0.033			0.651
Yes	2 (1.46%)	9 (6.47%)		2 (2.02%)	3 (3.03%)	
No	135 (98.54%)	130 (93.53%)		97 (97.98%)	96 (96.97%)	

Microvascular invasion			0.393			0.774
Yes	9 (6.57%)	13 (9.35%)		7 (7.07%)	6 (6.06%)	
No	128 (93.43%)	126 (90.65%)		92 (92.93%)	93 (93.94%)	

Hospital stay (day)	7.61 ± 3.86	10.70 ± 9.29	<0.001	7.85 ± 4.1	10.77 ± 10.03	0.008

HBV: hepatitis B virus, RBC: red blood cell, HB: hemoglobin, ALT: alanine aminotransferase, AST: aspartate transaminase, ALB: albumin, TB: total bilirubin, BUN: blood urea nitrogen, CR: creatinine, PT: prothrombin time, PTA: prothrombin activity, APTT: activated partial thromboplastin time, AFP: alpha-fetoprotein, CA19-9: carbohydrate antigen 19–9, CEA: carcinoembryonic antigen, IVC: inferior vena cava, and PVTT: portal vein tumor thrombosis.

**Table 2:** Postoperative complications of AR and NAR groups after PSM.

	NAR (*n* = 99)	AR (*n* = 99)	*p*
Complications (*n* (%))	15 (15.15%)	12 (12.12%)	0.534
Fever	3 (3.03%)	2 (2.02%)	0.882
Abdominal infection	1 (1.01%)	0	
Incision infection	1 (1.01%)	0	
Pulmonary infection	2 (2.02%)	1 (1.01%)	
Intestinal obstruction	1 (1.01%)	0	
Pleural effusion	0	1 (1.01%)	
Ascites	3 (1.01%)	2 (2.02%)	
Nausea or vomiting	1 (1.01%)	2 (2.02%)	
Thrombosis	1 (1.01%)	1 (1.01%)	
Bile or pancreatic leakage	1 (1.01%)	1 (1.01%)	
Hemorrhage	1 (1.01%)	2 (2.02%)	

Clavien–Dindo grade (*n* (%))			
I	12 (12.12%)	10 (10.10%)	0.825
II	3 (3.03%)	2 (2.02%)	

**Table 3:** Postoperative recurrence of AR and NAR groups after PSM.

	NAR (*n* = 99)	AR (*n* = 99)	*p*
Follow-up (*n* (%))			
Recurrence	84 (84.85%)	83 (83.84%)	0.120
Recurrence-free	1 (1.01%)	6 (6.06%)	
Lost during follow-up	14 (14.14%)	10 (10.10%)	

Intrahepatic recurrence (*n* (%))	68 (68.69%)	67 (67.68%)	0.879
Resection margin	5 (5.05%)	2 (2.02%)	0.245
Adjacent segment	32 (32.32%)	26 (26.26%)	
Distant segment	31 (31.31%)	39 (39.39%)	

Extrahepatic recurrence (*n* (%))	8 (8.08%)	11 (11.11%)	0.469
Single metastasis	3 (3.03%)	6 (6.06%)	0.463
Multiple metastases	5 (5.05%)	5 (5.05%)	

Both intra-/extra-hepatic recurrence (*n* (%))	8 (8.08%)	5 (5.05%)	0.389

**Table 4:** Univariate and multivariate analyses of relative risk of overall survival in the PSM cohort.

Variables	Univariate analysis		Multivariate analysis	
	HR (95%CI)	*p*	HR (95%CI)	*p*
Group (NAR vs. AR)	0.661 (0.460–0.949)	0.025	0.655 (0.448–0.958)	0.029
Age (year) (≤60 vs. >60)	1.089 (0.748–1.585)	0.657		
Sex (female vs. male)	0.921 (0.641–1.323)	0.655		
HBV infection (no vs. yes)	0.820 (0.527–1.275)	0.378		
Smoke history (no vs. yes)	0.913 (0.613–1.359)	0.652		
Alcohol history (no vs. yes)	0.959 (0.587–1.567)	0.868		
Child–Pugh classification (A vs. B)	0.832 (0.467–1.482)	0.533		
ALT (U/L) (≤40 vs. >40)	0.809 (0.517–1.265)	0.352		
AST (U/L) (≤40 vs. >40)	1.040 (0.678–1.598)	0.856		
ALB (g/L) (≤35 vs. >35)	0.651 (0.409–1.036)	0.070		
TB (*μ*mol/L) (≤20.5 vs. >20.5)	1.123 (0.731–1.725)	0.596		
CA19-9 (U/mL) (≤37 vs. >37)	1.273 (0.890–1.822)	0.186		
CEA (ng/mL) (≤20 vs. >20)	0.849 (0.414–1.740)	0.654		
AFP (ng/mL) (≤40 vs. >40)	0.668 (0.211–2.108)	0.491		
Cirrhosis (no vs. yes)	1.087 (0.684–1.728)	0.724		
Laparoscopic approach (no vs. yes)	0.618 (0.378–1.011)	0.055		
Robotic approach (no vs. yes)	0.547 (0.174–1.722)	0.302		
Portal vein occlusion (no vs. yes)	1.325 (0.919–1.909)	0.131		
IVC occlusion (no vs. yes)	0.999 (0.440–2.272)	0.999		
Intraoperative transfusion (no vs. yes)	1.577 (0.867–2.869)	0.136		
Conversion to open surgery (no vs. yes)	1.086 (0.568–2.075)	0.804		
Tumor size (cm) (≤5 vs. >5)	2.399 (1.666–3.453)	<0.001	2.050 (1.393–3.018)	<0.001
Tumor number (singular vs. multiple)	2.573 (1.754–3.775)	<0.001	1.962 (1.281–3.006)	0.002
Differentiation (poor vs. well/moderate)	0.323 (0.213–0.489)	<0.001	0.399 (0.261–0.609)	<0.001
Lymph node metastasis (no vs. yes)	1.759 (1.193–2.593)	0.004	0.983 (0.629–1.537)	0.942
Neuron invasion (no vs. yes)	0.552 (0.175–1.738)	0.310		
Necrosis in pathology (no vs. yes)	2.568 (0.941–7.006)	0.065		
PVTT in pathology (no vs. yes)	2.198 (0.891–5.418)	0.087		
Microvascular invasion (no vs. yes)	1.725 (0.967–3.075)	0.065		

**Table 5:** Univariate and multivariate analyses of relative risk of disease-free survival in the PSM cohort.

Variables	Univariate analysis		Multivariate analysis	
	HR (95%CI)	*p*	HR (95%CI)	*p*
Group (NAR vs. AR)	0.716 (0.526–0.974)	0.034	0.661 (0.476–0.916)	0.013
Age (year) (≤60 vs. >60)	0.975 (0.705–1.349)	0.880		
Sex (female vs. male)	0.955 (0.701–1.301)	0.770		
HBV infection (no vs. yes)	0.804 (0.563–1.148)	0.230		
Alcohol history (no vs. yes)	0.811 (0.530–1.242)	0.335		
Child–Pugh classification (A vs. B)	0.992 (0.583–1.689)	0.977		
Smoke history (no vs. yes)	0.932 (0.664–1.307)	0.682		
ALT (U/L) (≤40 vs. >40)	0.799 (0.550–1.160)	0.238		
AST (U/L) (≤40 vs. >40)	0.994 (0.692–1.427)	0.972		
ALB (g/L) (≤35 vs. >35)	0.677 (0.455–1.007)	0.054		
TB (*μ*mol/L) (≤20.5 vs. >20.5)	0.912 (0.620–1.341)	0.639		
CA19-9 (U/mL) (≤37 vs. >37)	1.028 (0.756–1.397)	0.860		
CEA (ng/mL) (≤20 vs. >20)	1.147 (0.662–1.985)	0.625		
AFP (ng/mL) (≤40 vs. >40)	0.961 (0.424–2.175)	0.924		
Cirrhosis (no vs. yes)	0.939 (0.623–1.418)	0.765		
Laparoscopic approach (no vs. yes)	0.550 (0.363–0.833)	0.005	0.790 (0.505–1.238)	0.304
Robotic approach (no vs. yes)	0.445 (0.165–1.202)	0.110		
Portal vein occlusion (no vs. yes)	1.261 (0.919–1.732)	0.151		
IVC occlusion (no vs. yes)	1.327 (0.699–2.521)	0.387		
Intraoperative transfusion (no vs. yes)	1.586 (0.945–2.660)	0.081		
Conversion to open surgery (no vs. yes)	0.874 (0.474–1.612)	0.667		
Tumor size (cm) (≤5 vs. >5)	1.963 (1.439–2.677)	<0.001	1.646 (1.170–2.315)	0.004
Tumor number (singular vs. multiple)	1.771 (1.229–2.553)	0.002	1.185 (0.784–1.185)	0.422
Differentiation (poor vs. well/moderate)	0.457 (0.331–0.633)	<0.001	0.577 (0.410–0.812)	0.002
Lymph node metastasis (no vs. yes)	1.987 (1.404–2.812)	<0.001	1.345 (0.898–2.014)	0.150
Neuron invasion (no vs. yes)	0.862 (0.404–1.840)	0.701		
Necrosis in pathology (no vs. yes)	2.462 (0.905–6.695)	0.078		
PVTT in pathology (no vs. yes)	1.457 (0.536–3.959)	0.461		
Microvascular invasion (no vs. yes)	1.315 (0.726–2.379)	0.366		

## Data Availability

The data for the current study are available from the corresponding authors on reasonable request.

## References

[1] Brindley P. J., Bachini M., Ilyas S. I. (2021). Cholangiocarcinoma. *Nature Reviews Disease Primers*.

[2] Izquierdo-Sanchez L. (2021). Cholangiocarcinoma landscape in Europe: diagnostic, prognostic and therapeutic insights from the ENSCCA registry. *Journal of Hepatology*.

[3] Banales J. M., Marin J. J. G., Lamarca A. (2020). Cholangiocarcinoma 2020: the next horizon in mechanisms and management. *Nature Reviews Gastroenterology & Hepatology*.

[4] Blechacz B., Komuta M., Roskams T., Gores G. J. (2011). Clinical diagnosis and staging of cholangiocarcinoma. *Nature Reviews Gastroenterology & Hepatology*.

[5] Soares K. C., Jarnagin W. R. (2021). The landmark series: hilar cholangiocarcinoma. *Annals of Surgical Oncology*.

[6] Cai J. Q., Cai S. W., Cong W. M. (2014). Diagnosis and treatment of cholangiocarcinoma: a consensus from surgical specialists of China. *Journal of Huazhong University of Science and Technology - Medical sciences*.

[7] Loeuillard E., Conboy C. B., Gores G. J., Rizvi S. (2019). Immunobiology of cholangiocarcinoma. *JHEP Reports*.

[8] Turgeon M. K., Maithel S. K. (2020). Cholangiocarcinoma: a site-specific update on the current state of surgical management and multi-modality therapy. *Chinese Clinical Oncology*.

[9] Bektas H., Yeyrek C., Kleine M. (2015). Surgical treatment for intrahepatic cholangiocarcinoma in Europe: a single center experience. *Journal of Hepato-Biliary-Pancreatic Sciences*.

[10] Zhu H., Wang L., Wang M. (2021). Prognostic value of resection margin length after surgical resection for intrahepatic cholangiocarcinoma. *The American Journal of Surgery*.

[11] Uenishi T., Yamamoto T., Takemura S., Kubo S. (2014). Surgical treatment for intrahepatic cholangiocarcinoma. *Clinical Journal of Gastroenterology*.

[12] Zhang H., Yang T., Wu M., Shen F. (2016). Intrahepatic cholangiocarcinoma: epidemiology, risk factors, diagnosis and surgical management. *Cancer Letters*.

[13] Kosaka H., Kaibori M., Matsui K., Ishizaki M., Matsushima H., Sekimoto M. (2021). Investigation of a tumor location-specific therapeutic strategy for intrahepatic cholangiocarcinoma. *Asian Pacific Journal of Cancer Prevention*.

[14] Makuuchi M., Hasegawa H., Yamazaki S. (1985). Ultrasonically guided subsegmentectomy. *Surgery Gynecology & Obstetrics*.

[15] Minagawa M., Mise Y., Omichi K. (2022). Anatomic resection for hepatocellular carcinoma: prognostic impact assessed from recurrence treatment. *Annals of Surgical Oncology*.

[16] Sun Z., Li Z., Shi X. L., He X. W., Chen J., Song J. H. (2021). Anatomic versus non-anatomic resection of hepatocellular carcinoma with microvascular invasion: a systematic review and meta-analysis. *Asian Journal of Surgery*.

[17] Okamura Y., Sugiura T., Ito T. (2021). Anatomical resection is useful for the treatment of primary solitary hepatocellular carcinoma with predicted microscopic vessel invasion and/or intrahepatic metastasis. *Surgery Today*.

[18] Si A., Li J., Yang Z. (2019). Impact of anatomical versus non-anatomical liver resection on short- and long-term outcomes for patients with intrahepatic cholangiocarcinoma. *Annals of Surgical Oncology*.

[19] Li B., Song J. L., Aierken Y., Chen Y., Zheng J. L., Yang J. Y. (2018). Nonanatomic resection is not inferior to anatomic resection for primary intrahepatic cholangiocarcinoma: a propensity score analysis. *Scientific Reports*.

[20] Hwang S., Lee Y. J., Song G. W. (2015). Prognostic impact of tumor growth type on 7th AJCC staging system for intrahepatic cholangiocarcinoma: a single-center experience of 659 cases. *Journal of Gastrointestinal Surgery*.

[21] Oguro S., Yoshimoto J., Imamura H., Ishizaki Y., Kawasaki S. (2018). Clinical significance of macroscopic no-margin hepatectomy for hepatocellular carcinoma. *International Hepato-Pancreato-Biliary Association*.

[22] Valle J. W., Borbath I., Khan S., Huguet F., Gruenberger T., Arnold D. (2016). Biliary cancer: ESMO clinical practice guidelines for diagnosis, treatment and follow-up. *Annals of Oncology*.

[23] Benson A. B., D’Angelica M. I., Abbott D. E. (2021). Hepatobiliary cancers, version 2.2021, NCCN clinical practice guidelines in oncology. *Journal of the National Comprehensive Cancer Network*.

[24] Casadio M., Cardinale V., Klumpen H. J. (2022). Setup of multidisciplinary team discussions for patients with cholangiocarcinoma: current practice and recommendations from the European Network for the Study of Cholangiocarcinoma (ENS-CCA). *ESMO Open*.

[25] Zhang B. (2022). Expert consensus on organizing the multidisciplinary team (MDT) diagnosis and treatment of hepato-pancreato-biliary diseases in China. *Science China. Life Sciences*.

[26] Dindo D., Demartines N., Clavien P. A. (2004). Classification of surgical complications: a new proposal with evaluation in a cohort of 6336 patients and results of a survey. *Annals of Surgery*.

[27] Rubin D. B., Thomas N. (1996). Matching using estimated propensity scores: relating theory to practice. *Biometrics*.

[28] Austin P. C. (2011). Optimal caliper widths for propensity-score matching when estimating differences in means and differences in proportions in observational studies. *Pharmaceutical Statistics*.

[29] Zori A. G., Yang D., Draganov P. V., Cabrera R. (2021). Advances in the management of cholangiocarcinoma. *World Journal of Hepatology*.

[30] Lauterio A., De Carlis R., Centonze L. (2021). Current surgical management of peri-hilar and intra-hepatic cholangiocarcinoma. *Cancers*.

[31] Zhang F., Lu C. D., Zhang X. P. (2020). The impact of portal vein tumor thrombus on long-term survival after liver resection for primary hepatic malignancy. *International Hepato-Pancreato-Biliary Association*.

[32] Takamoto T., Makuuchi M. (2019). Precision surgery for primary liver cancer. *Cancer Biology & Medicine*.

[33] Zhang B., Dong W., Luo H. (2016). Surgical treatment of hepato-pancreato-biliary disease in China: the Tongji experience. *Science China Life Sciences*.

[34] Lendoire J. C., Gil L., Imventarza O. (2018). Intrahepatic cholangiocarcinoma surgery: the impact of lymphadenectomy. *Chinese Clinical Oncology*.

[35] Czauderna C., Kirstein M. M., Tews H. C., Vogel A., Marquardt J. U. (2021). Molecular subtypes and precision oncology in intrahepatic cholangiocarcinoma. *Journal of Clinical Medicine*.

[36] Cillo U., Fondevila C., Donadon M. (2019). Surgery for cholangiocarcinoma. *Liver International*.

[37] Seo J. W., Kwan B. S., Cheon Y. K. (2020). Prognostic impact of hepatitis B or C on intrahepatic cholangiocarcinoma. *Korean Journal of Internal Medicine (Korean Edition)*.

[38] Zhang B., Zhang B., Zhang Z. (2018). 42, 573 cases of hepatectomy in China: a multicenter retrospective investigation. *Science China Life Sciences*.

[39] Ahn C. S., Hwang S., Lee Y. J. (2018). Prognostic impact of hepatitis B virus infection in patients with intrahepatic cholangiocarcinoma. *ANZ Journal of Surgery*.

[40] Liu R. Q., Shen S. J., Hu X. F., Liu J., Chen L. J., Li X. Y. (2013). Prognosis of the intrahepatic cholangiocarcinoma after resection: hepatitis B virus infection and adjuvant chemotherapy are favorable prognosis factors. *Cancer Cell International*.

[41] Cloyd J. M., Ejaz A., Pawlik T. M. (2020). The landmark series: intrahepatic cholangiocarcinoma. *Annals of Surgical Oncology*.

[42] Ben Khaled N. (2021). Current state of multidisciplinary treatment in cholangiocarcinoma. *Digestive Diseases (Basel, Switzerland)*.

[43] Michalopoulos G. K. (2014). Advances in liver regeneration. *Expert Review of Gastroenterology & Hepatology*.

[44] Li M. X., Bi X. Y., Li Z. Y. (2016). Impaction of surgical margin status on the survival outcome after surgical resection of intrahepatic cholangiocarcinoma: a systematic review and meta-analysis. *Journal of Surgical Research*.

[45] Zhou R., Lu D., Li W. (2019). Is lymph node dissection necessary for resectable intrahepatic cholangiocarcinoma? A systematic review and meta-analysis. *International Hepato-Pancreato-Biliary Association*.

[46] Di Martino M. (2022). It is the lymph node ratio that determines survival and recurrence patterns in resected distal cholangiocarcinoma. A multicenter international study. *European Journal of Surgical Oncology*.

[47] Lu W., Tang Z. H., Quan Z. W. (2019). Viewpoint of systematic lymphadenectomy for intrahepatic cholangiocarcinoma patients. *Zhonghua Wai Ke Za Zhi*.

[48] Umeda Y., Mitsuhashi T., Kojima T. (2022). Impact of lymph node dissection on clinical outcomes of intrahepatic cholangiocarcinoma: inverse probability of treatment weighting with survival analysis. *Journal of Hepato-Biliary-Pancreatic Sciences*.

[49] Rodrigues P. M., Vogel A., Arrese M., Balderramo D. C., Valle J. W., Banales J. M. (2021). Next-generation biomarkers for cholangiocarcinoma. *Cancers*.

[50] Ji J., Liu L., Jiang F. (2021). The clinical application of PIVKA-II in hepatocellular carcinoma and chronic liver diseases: a multi-center study in China. *Journal of Clinical Laboratory Analysis*.

[51] Yanagaki M., Shirai Y., Hamura R. (2022). Novel combined fibrosis-based index predicts the long-term outcomes of hepatocellular carcinoma after hepatic resection. *International Journal of Clinical Oncology*.

[52] Qiu Y., He J., Chen X., Huang P., Hu K., Yan H. (2018). The diagnostic value of five serum tumor markers for patients with cholangiocarcinoma. *Clinica Chimica Acta*.

[53] Tian M., Liu W., Tao C. (2020). Prediction of overall survival in resectable intrahepatic cholangiocarcinoma: IS (ICC) -applied prediction model. *Cancer Science*.

[54] Krenzien F., Nevermann N., Krombholz A. (2022). Treatment of intrahepatic cholangiocarcinoma-A multidisciplinary approach. *Cancers*.

[55] Lang S. A., Bednarsch J., Joechle K. (2021). Prognostic biomarkers for cholangiocarcinoma (CCA): state of the art. *Expert Review of Gastroenterology & Hepatology*.

[56] Yamashita Y. i., Taketomi A., Morita K. (2008). The impact of surgical treatment and poor prognostic factors for patients with intrahepatic cholangiocarcinoma: retrospective analysis of 60 patients. *Anticancer Research*.

[57] Kong J., Cao Y., Chai J. (2020). Effect of tumor size on long-term survival after resection for solitary intrahepatic cholangiocarcinoma. *Frontiers Oncology*.

[58] Yu T. H., Chen X., Zhang X., Zhang E., Sun C. (2021). Clinicopathological characteristics and prognostic factors for intrahepatic cholangiocarcinoma: a population-based study. *Scientific Reports*.

[59] Wang Y., Li J., Xia Y. (2013). Prognostic nomogram for intrahepatic cholangiocarcinoma after partial hepatectomy. *Journal of Clinical Oncology*.

[60] Hyder O., Marques H., Pulitano C. (2014). A nomogram to predict long-term survival after resection for intrahepatic cholangiocarcinoma: an Eastern and Western experience. *JAMA Surgery*.

[61] Wang M., Gao Y., Feng H. (2018). A nomogram incorporating six easily obtained parameters to discriminate intrahepatic cholangiocarcinoma and hepatocellular carcinoma. *Cancer Medicine*.

[62] Regmi P., Hu H. J., Paudyal P. (2021). Is laparoscopic liver resection safe for intrahepatic cholangiocarcinoma? A meta-analysis. *European Journal of Surgical Oncology*.

